# Draft Genome Sequence of *Kurthia huakuii* LAM0618^T^, an Organic-Pollutant-Degrading Strain Isolated from Biogas Slurry

**DOI:** 10.1128/genomeA.01158-13

**Published:** 2014-01-16

**Authors:** Zhiyong Ruan, Yanwei Wang, Jinlong Song, Yi Zhai, Chi Zhang, Chao Chen, Yanting Li, Bingqiang Zhao, Bin Zhao

**Affiliations:** Key Laboratory of Microbial Resources (Ministry of Agriculture, China), Institute of Agricultural Resources and Regional Planning, CAAS, Beijing, Chinaa; Institute of Microbiology, Chinese Academy of Sciences, Beijing, Chinab; Agricultural Engineering Institute, Chongqing Academy of Agricultural Sciences, Chongqing, Chinac; State Key Laboratory of Agricultural Microbiology, College of Life Science and Technology, Huazhong Agricultural University, Wuhan, Chinad

## Abstract

*Kurthia huakuii* LAM0618^T^ is a facultative anaerobic pollutant-degrading bacterium isolated from biogas slurry. An analysis of the draft genome sequence of LAM0618^T^ reveals a genome size of 3,585,165 bp, with a mean G+C content of 39.1%. The genome contains 3,560 coding sequences and 112 tRNA and 33 rRNA genes.

## GENOME ANNOUNCEMENT

*Kurthia huakuii* LAM0618^T^ (ACCC 06121^T^ = JCM 19187^T^), a Gram-positive, motile, non-spore-forming, short-rod-shaped, and facultative anaerobic bacterium isolated from biogas slurry (1), was found to be capable of degrading sulfonylurea herbicides (2), acetochlor, and alkanes and decolorizing triphenylmethane dyes, and it may provide new insights into bioremediation applications.

The genome of *K. huakuii* LAM0618^T^ was sequenced using the Illumina HiSeq 2000 platform with a whole-genome shotgun (WGS) strategy at the Beijing Genomics Institute (BGI) (Shenzhen, China), and 9,345,778 reads totaling 755 Mb of draft data were generated. All clean reads of 3,585,165 bp were assembled into 56 contigs and 25 scaffolds using SOAP*denovo* version 2.04 (3) (http://sourceforge.net/projects/soapdenovo2/files/SOAPdenovo2/). Open reading frames (ORFs) were identified by Glimmer version 3.02 (4) (http://www.cbcb.umd.edu/software/glimmer/). The functions of these ORFs were further annotated by comparing with the KEGG, COG, GO, NR, and Swiss-Prot databases. The rRNAs were predicted by RNAmmer version 1.2 (5), and tRNAs were predicted by tRNAscan-SE version 1.23 (6), based on the hits to the Rfam database. The G+C content of the genome is 39.1%, and it contains 3,560 coding sequences (CDSs), 112 tRNA genes, and 33 rRNA genes. A total of 2,242 protein-coding genes were assigned a predicted function and have been categorized into 21 COG groups.

An analysis of the genome of *K. huakuii* LAM0618^T^ may contribute to the identification of diverse biodegradation genes and further, to elucidation of the microbial degradation pathways for sulfonylurea herbicides and other organic pollutants.

### Nucleotide sequence accession numbers.

The draft genome sequence of *K. huakuii* LAM0618^T^ has been deposited in DDBJ/EMBL/GenBank under the accession no. AYTB00000000. The version described in this paper is version AYTB01000000.
